# Supplementary material for: In Silico Examination of Single Nucleotide Missense Mutations in NHLH2, a Gene Linked to Infertility and Obesity
Source: Int J Mol Sci. 2023 Feb 6;24(4):3193. doi: 10.3390/ijms24043193 (PMC9968165; doi:10.3390/ijms24043193)
Supplement: Supplementary file 1 [file ijms-24-03193-s001.zip › Supplemental Figure 1.pdf]

Supplemental Figure S1. SNAP heatmap of single nucleotide variants in *NHLH2*. The SNAP database (1) was used for prediction, and the heatmap generated for each position in the protein. Red intensity increases with increased predicted effect of the variant. Blue squares predict changes that are neutral, while black squares are the amino acid in the normal sequence. Arrowheads point to the 38 variants identified by PROVEAN (2)and used in our analysis

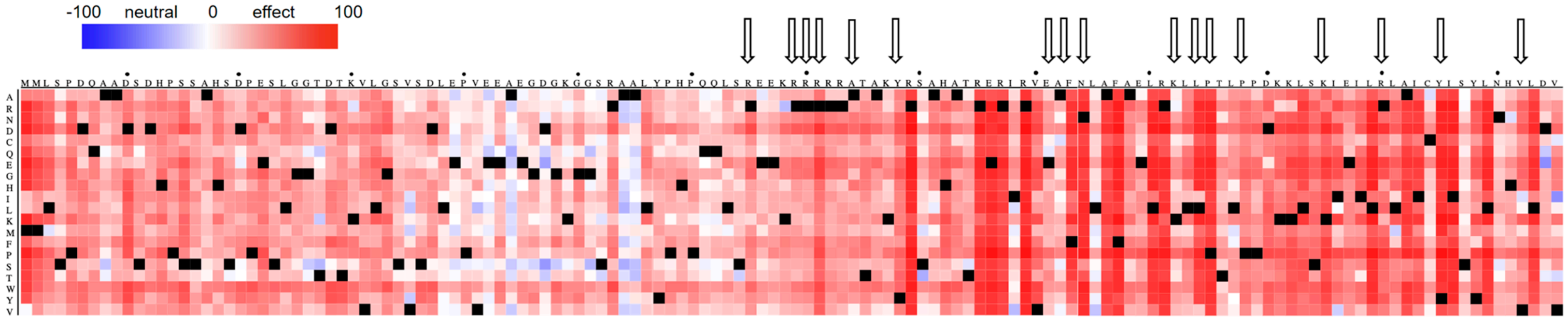

<u>References</u>

1. Bromberg Y, Rost B. SNAP: predict effect of non-synonymous polymorphisms on function. Nucleic Acids Res. 2007;35(11):3823-35.
2. Choi Y, Chan AP. PROVEAN web server: a tool to predict the functional effect of amino acid substitutions and indels. Bioinformatics. 2015;31(16):2745-7.
