# Supplementary material for: In Silico Examination of Single Nucleotide Missense Mutations in NHLH2, a Gene Linked to Infertility and Obesity
Source: Int J Mol Sci. 2023 Feb 6;24(4):3193. doi: 10.3390/ijms24043193 (PMC9968165; doi:10.3390/ijms24043193)
Supplement: Supplementary file 1 [file ijms-24-03193-s001.zip › Supplemental Table S1.pdf]

Supplemental Table S1. Analysis of Pathogenic Missense Variants in *NHLH2*. The reference SNP (rs) number hyperlinked to the NCBI dbSNP database (1) is provided for each variant. Amino acid changes, and frequency for each variant was taken from the dbSNP listing. PROVEAN Score (2)was calculated for all 318 *NHLH2* missense variants, and those with a score of below -2.500 are listed here. PROVEAN score higher than -2.500 are considered to have lower pathogenicity. Mutation assessor Function Impact (FI) scores are provided. FI scores of ≤ 0.8 are considered neutral, 0.8-1.9 are considered low impact (italics), ≥1.9-3.5 are considered medium impact (underline), and >3.5 are considered high impact (bold). Next, the "Screening for Non-Acceptable Polymorphisms" (SNAP) tool scores (3) are provided, with scores >50 indicating strong signal for effect (bold), -50 <score<50, indicating weak signal for effect (italics), and score < -50 indicating neutral or no effect. None of the SNVs listed here were in the neutral category. The "Cancer Related Analysis of Variants Toolkit" (CRAVAT) score (4) is provided in the last column with *P*-values given for scores indicating deleterious variants (bold). All others were considered neutral. One variant was not predictable using the CRAVAT toolkit and is marked n/a.

RS #	Amino acid Alteration	Frequency	PROVEAN Score	Mutation Assessor FI Score	SNAP Score	CRAVAT Score
rs372688621	R65L<br>R65H	C>A / C>T<br>A=0.000007 (1/140238, GnomAD)<br>A=0.00008 (1/12086, GO-ESP)<br>A=0.00000 (0/10680, ALFA)	-3.844 (R65L)<br>-3.360 (R65H)	<u>2.125</u><br><u>2.123</u>	*37*<br>*37*	0.311<br>0.271
rs1194455186	R65S	G>T<br>T=0.000004 (1/264690, TOPMED)<br>T=0.000007 (1/140248, GnomAD)<br>T=0.00000 (0/14050, ALFA)	-3.624	<u>2.125</u>	*41*	0.301
rs776027891	E66V	T>A<br>A=0.00000 (0/41790, ExAC)	-4.283	<u>2.125</u>	*9*	0.324
rs765797948	R69L	C>A<br>A=0.00002 (1/45328, ExAC)	-5.583	<u>2.125</u>	*31*	0.373
rs1262624693	R70G	G>C<br>C=0.000004 (1/264690, TOPMED)<br>C=0.00000 (0/14050, ALFA)<br>C= 0.000015 (1/197814, All of Us)	-5.489	<u>2.125</u>	**56**	0.350
rs1417094020	R71H	C>T<br>T=0.000005 (1/194090, GnomAD_exome)<br>T=0.000007 (1/140262, GnomAD)<br>T=0.00000 (0/10680, ALFA)	-3.808	<u>2.085</u>	**57**	0.301
rs1650933387	R71G	G>C<br>C=0.00005 (1/20978, ALFA)	-4.906	<u>2.085</u>	**73**	0.345
rs772525034	A74P	C>A / C>G<br>A=0.000005 (1/204030, GnomAD_exome)	-3.421	<u>2.125</u>	*50*	0.338
rs1199787521	Y78H	A>G<br>No frequency information	-3.871	*1.15*	**53**	0.334
rs1650932250	Y78C	T>C<br>C=0.00000 (0/10680, ALFA)	-7.07	*1.15*	**54**	0.562
rs1368574494	A83T	C>T<br>No frequency information	-2.706	-0.49	*35*	0.535
rs1650931436	R89L	C>A<br>A=0.000004 (1/264690, TOPMED)<br>A=0.00000 (0/10680, ALFA)	-6.9	**4.48**	**83**	**0.918**<br>p = 0.008
rs1650931347	E91K	C>T<br>T=0.00000 (0/10680, ALFA)	-3.203	-0.39	*34*	0.492

rs199738358	A92T	C>T<br>T=0.000008 (2/264690, TOPMED)<br>T=0.000013 (3/238512, GnomAD_exome)<br>T=0.000007 (1/140296, GnomAD)	-2.648	-0.83	*17*	0.453
rs1352643678	N94T	T>G<br>G=0.000008 (2/240462, GnomAD_exome)	-6	<u>2.895</u>	**65**	**0.749**<br>p = 0.050
rs867911589	R101H	C>T<br>No frequency information	-4.938	**4.03**	**73**	0.525
rs781142041	K102T	T>G<br>G=0.000008 (2/243050, GnomAD_exome)<br>G=0.000018 (2/110334, ExAC)	-4.955	*1.68*	*10*	0.200
rs757420009	L104R	A>C<br>C=0.000004 (1/243140, GnomAD_exome)<br>C=0.000009 (1/111870, ExAC)	-5.8	**3.695**	**81**	**0.904**<br>p = 0.010
rs1650929924	P105S	G>A<br>A=0.000004 (1/264690, TOPMED)<br>A=0.00000 (0/10680, ALFA)	-8	<u>3.355</u>	**77**	**0.810**<br>p = 0.028
rs751807396	P108T	G>T<br>T=0.000000 (0/113014, ExAC)	-7.733	<u>2.125</u>	*47*	0.550
rs1282822521	P109A	G>C<br>C=0.000004 (1/243776, GnomAD_exome)	-6.967	*1.38*	*21*	0.258
rs1650929118	L113F	G>A<br>A=0.000007 (1/140298, GnomAD)<br>A=0.00000 (0/10680, ALFA)	-3.733	**3.775**	**54**	**0.878**<br>p = 0.014
rs1650928889	S114Y	G>T<br>T=0.000004 (1/264690, TOPMED)<br>T=0.00000 (0/10680, ALFA)	-5.567	**4.365**	**59**	**0.885**<br>p = 0.013
rs1650928951	S114P	A>G<br>G=0.000004 (1/264690, TOPMED)<br>G=0.00000 (0/10680, ALFA)	-4.3	<u>2.545</u>	**58**	**0.870**<br>p = 0.015
rs1354640857	K115N	C>A<br>A=0.0004 (2/4480, Estonian)<br>A=0.0004 (2/4470, ALFA)	-4.633	**3.515**	**70**	**0.946**<br>p = 0.004
rs1313234520	E117A	T>G<br>G=0.000004 (1/264690, TOPMED)<br>G=0.000007 (1/140270, GnomAD)<br>G=0.00000 (0/10680, ALFA)<br>G= 0.000005 (1/197178, All of Us)	-5.633	<u>2.245</u>	*28*	**0.835**<br>p = 0.022
rs1650928729	E117Q	C>G<br>G=0.000007 (1/140294, GnomAD)<br>G=0.00000 (0/10680, ALFA)<br>G= 0.000010/197180 (All of Us)	-2.8	<u>2.455</u>	*22*	0.742
rs1650928569	I118T	A>G<br>G=0.000004 (1/264690, TOPMED)<br>G=0.00000 (0/10680, ALFA)	-4.2	0.45	*7*	0.371
rs1557829654	R120P	C>G<br>G=0.000004 (1/244856, GnomAD_exome)	-6.019	**3.875**	**77**	**0.881**<br>p = 0.013
rs1650928263	R120S	G>T<br>No frequency information	-5.019	**3.875**	**57**	**0.856**<br>p = 0.017

rs866172895	A122T	C>T<br>T=0.000 (0/646, ALFA)	-3.585	*1.68*	**55**	**0.922**<br>p = 0.007
rs1436582067	A122V	G>A<br>A=0.000007 (1/140296, GnomAD)<br>A=0.00000 (0/10680, ALFA)<br>A= 0.000005/197174 (All of Us)	-3.681	2.875	**67**	**0.943**<br>p = 0.005
rs759974809	I123T	A>G<br>G=0.000009 (1/112944, ExAC)	-4.013	*1.865*	**61**	0.358
rs1433737875	Y125C	T>C<br>C=0.000007 (1/140280, GnomAD)<br>C= 0.000005 (1/197174, All of Us)	-8.367	**4.08**	**68**	**0.960**<br>p = 0.003
rs761527985	H131Q	G>A / G>T<br>A=0.00001 (1/99560, ExAC)<br>A=0.0006 (1/1774, Korea1K)	-4.781	-0.375	*24*	n/a
rs1230535357	V132F	C>A<br>A=0.000004 (1/264690, TOPMED)<br>A=0.000007 (1/140280, GnomAD)<br>A=0.00000 (0/14050, ALFA)	-3.496	*1.465*	*41*	0.718

References

1. Kitts A, Phan L, Ward M, Holmes JB. T (dbSNP). In: (US) NCfBI, editor. The NCBI Handbook [Internet] 2nd edition. Bethesda, MD2013. p. https://www.ncbi.nlm.nih.gov/books/NBK174586/.

2. Choi Y, Chan AP. PROVEAN web server: a tool to predict the functional effect of amino acid substitutions and indels. Bioinformatics. 2015;31(16):2745-7.

3. Bromberg Y, Rost B. SNAP: predict effect of non-synonymous polymorphisms on function. Nucleic Acids Res. 2007;35(11):3823-35.

4. Masica DL, Douville C, Tokheim C, Bhattacharya R, Kim R, Moad K, et al. CRAVAT 4: Cancer-Related Analysis of Variants Toolkit. Cancer Res. 2017;77(21):e35-e8.
