# Supplementary material for: In Silico Examination of Single Nucleotide Missense Mutations in NHLH2, a Gene Linked to Infertility and Obesity
Source: Int J Mol Sci. 2023 Feb 6;24(4):3193. doi: 10.3390/ijms24043193 (PMC9968165; doi:10.3390/ijms24043193)
Supplement: Supplementary file 1 [file ijms-24-03193-s001.zip › Supplemental Table S2.pdf]

Supplemental Table S2: DNA binding prediction using IntFOLD (1). The IntFOLD and the associated FunFOLD servers were used to model NHLH2 protein containing each of the 37 variants (1, 2). Tertiary models were captured from the left side (left picture) and from the top view (right picture). In some cases, the DNA binding prediction appears altered compared to the WT NHLH2 prediction and was marked as such in column 3.

**SNV**	**Tertiary structure with DNA**	**DNA binding prediction**
Rs372688621<br><br>R65L<br>R65H	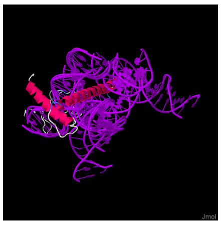 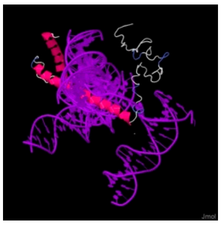<br>R65L<br><br>R65H: no model	R65L: Binds DNA (altered)<br><br>R65H: not predicted to bind DNA
Rs1194455186<br><br>R65S	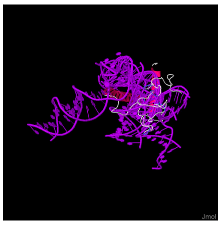 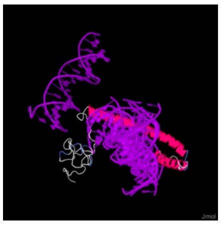	Binds DNA (altered)
Rs776027891<br><br>E66V	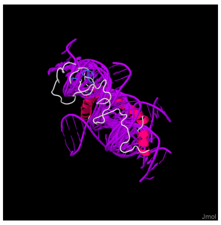 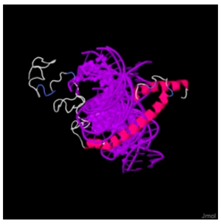	Binds DNA
Rs765797948<br><br>R69L	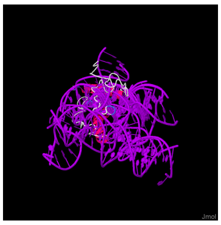 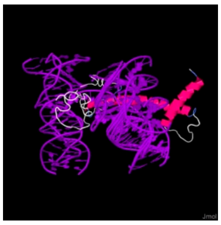	Binds DNA
Rs1262624693<br><br>R70G	No model	Not predicted to bind DNA

Rs1417094020<br><br>R71H	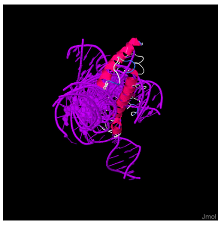 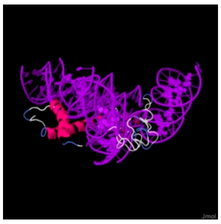	Binds DNA
Rs1650933387<br><br>R71G	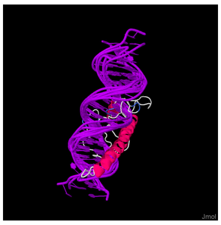 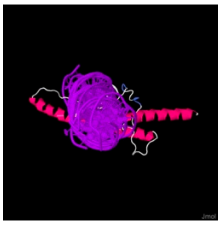	Binds DNA
Rs772525034<br><br>A74P	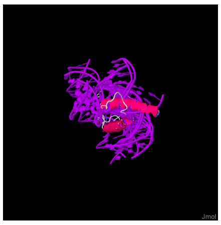 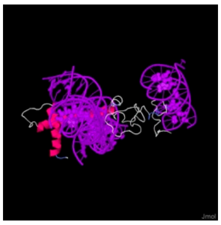	Binds DNA (altered)
Rs1199787521<br><br>Y78H	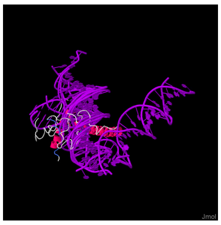 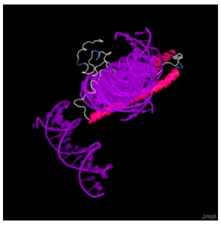	Binds DNA (altered)
Rs1650932250<br><br>Y78C	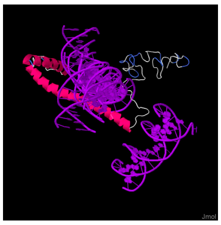 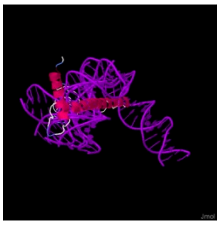	Binds DNA (altered)
Rs1368574494<br><br>A83T	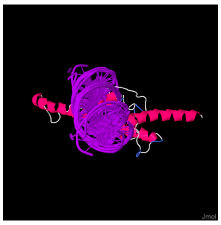 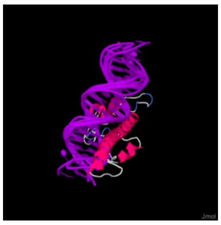	Binds DNA

Rs1650931436<br><br>R89L	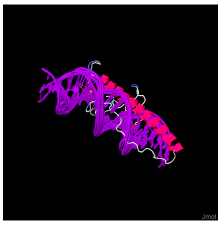 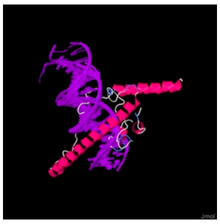	Binds DNA
Rs1650931347<br><br>E91K	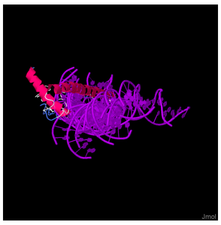 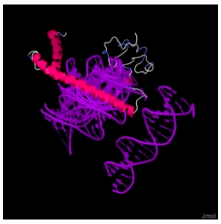	Binds DNA (altered)
Rs199738358<br><br>A92T	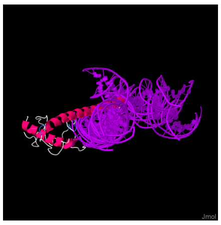 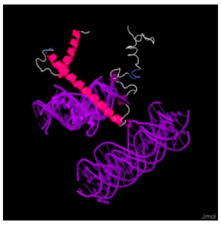	Binds DNA (altered)
Rs1352643678<br><br>N94T	No model	Not predicted to bind DNA
Rs867911589<br><br>R101H	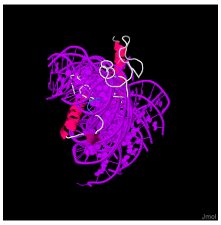 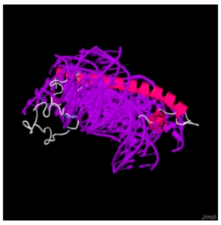	Binds DNA
Rs781142041<br><br>K102T	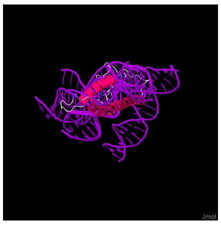 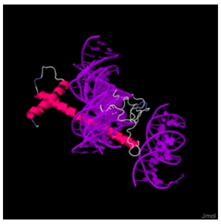	Binds DNA (altered)
Rs757420009<br><br>L104R	No model	not predicted to bind DNA

Rs1650929924<br><br>P105S	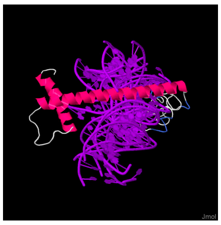 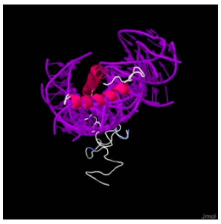	Binds DNA
Rs751807396<br><br>P108T	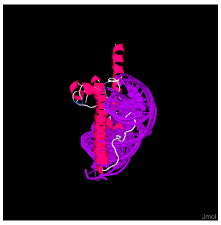 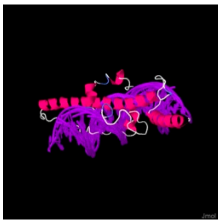	Binds DNA
Rs1282822521<br><br>P109A	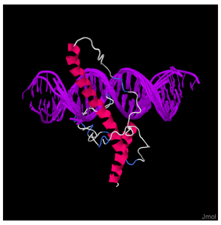 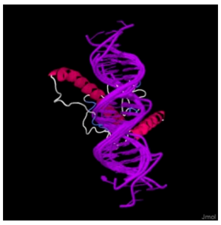	Binds DNA
Rs1650929118<br><br>L113F	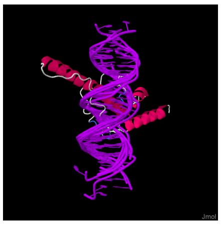 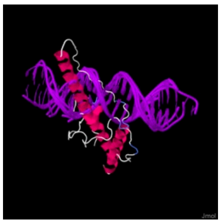	Binds DNA
Rs1650928889<br><br>S114Y	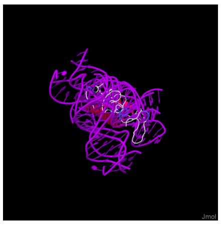 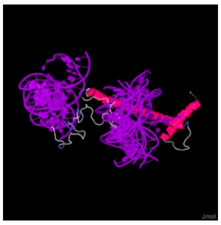	Binds DNA
Rs1650928951<br><br>S114P	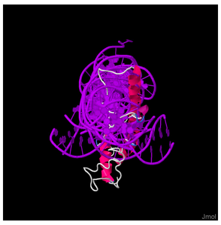 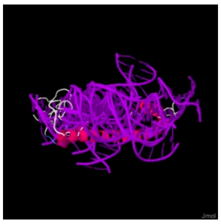	Binds DNA
Rs1354640857<br><br>K115N	No model	Not predicted to bind DNA

Rs1313234520<br>E117A	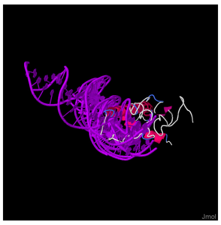 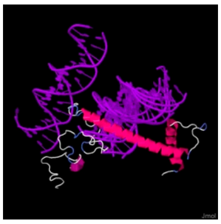	Binds DNA (altered)
E117Q	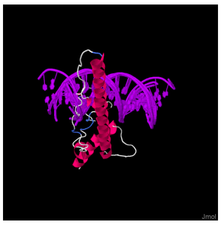 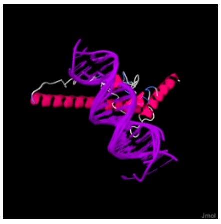	Binds DNA
I118T	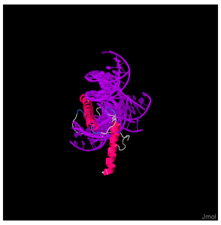 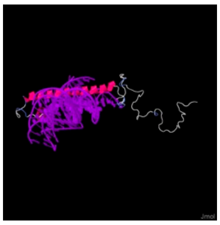	Binds DNA
Rs1557829654<br>R120P	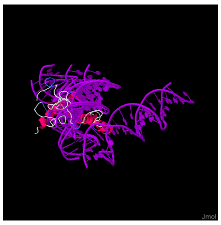 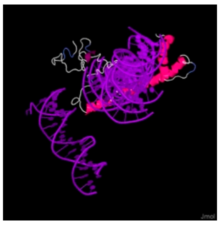	Binds DNA (altered)
Rs1650928263<br>R120S	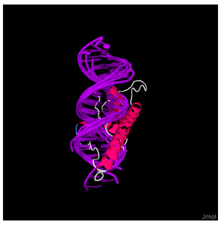 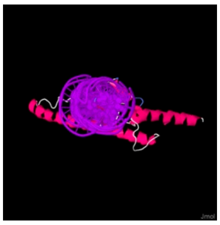	Binds DNA
Rs866172895<br>A122T	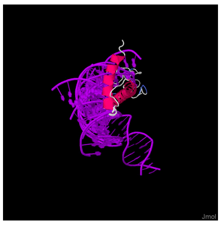 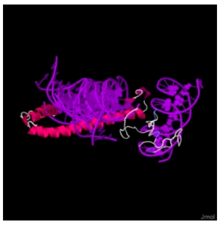	Binds DNA

Rs1436582067<br><br>A122V	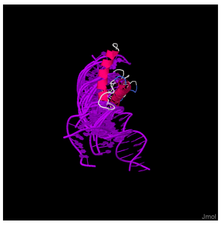 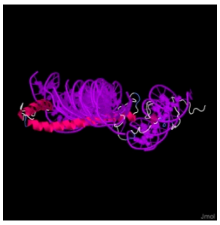	Binds DNA
Rs759974809<br><br>I123T	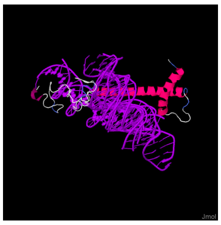 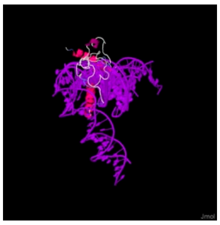	Binds DNA
Rs1433737875<br><br>Y125C	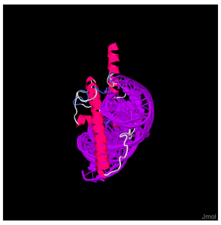 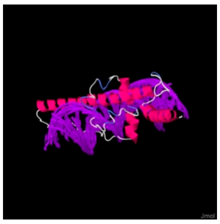	Binds DNA
Rs761527985<br><br>H131Q	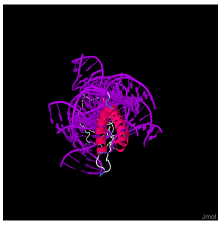 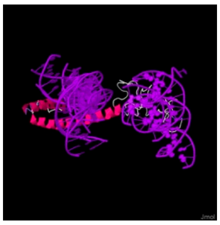	Binds DNA
Rs1230535357<br><br>V132F	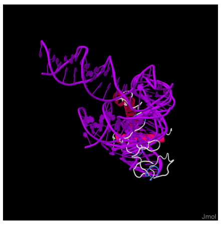 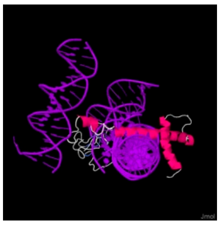	Binds DNA (altered)

References

1. McGuffin LJ, Adiyaman R, Maghrabi AHA, Shuid AN, Brackenridge DA, Nealon JO, et al. IntFOLD: an integrated web resource for high performance protein structure and function prediction. Nucleic Acids Res. 2019;47(W1):W408-W13.
2. Roche DB, Buenavista MT, McGuffin LJ. The FunFOLD2 server for the prediction of protein-ligand interactions. Nucleic Acids Res. 2013;41(Web Server issue):W303-7.
